# Prenatal hepatitis C screening, diagnoses, and follow-up testing in British Columbia, 2008–2019

**DOI:** 10.1371/journal.pone.0244575

**Published:** 2020-12-31

**Authors:** Margo E. Pearce, Amanda Yu, Maria Alvarez, Sofia R. Bartlett, Mawuena Binka, Dahn Jeong, Emilia Clementi, Prince Adu, James Wilton, Eric M. Yoshida, Neora Pick, Jane A. Buxton, Jason Wong, Agatha Jassem, Mel Krajden, Naveed Z. Janjua

**Affiliations:** 1 British Columbia Centre for Disease Control, Vancouver, British Columbia, Canada; 2 School of Population and Public Health, University of British Columbia, Vancouver, British Columbia, Canada; 3 Department of Pathology and Laboratory Medicine, University of British Columbia, Vancouver, British Columbia, Canada; 4 Department of Medicine, Division of Gastroenterology, University of British Columbia, Vancouver, British Columbia, Canada; 5 Vancouver General Hospital, Vancouver, British Columbia, Canada; 6 Department of Medicine, Division of Infectious Diseases, University of British Columbia Vancouver, British Columbia, Canada; 7 British Columbia Women’s Hospital, Vancouver, British Columbia, Canada; Centers for Disease Control and Prevention, UNITED STATES

## Abstract

**Objective:**

Current guidelines in British Columbia recommend prenatal screening for hepatitis C antibodies (anti-HCV) if risk factors are present. We aimed to estimate frequency of prenatal anti-HCV testing, new diagnoses, repeated and follow-up testing among BC women.

**Methods:**

BC Centre for Disease Control Public Health Laboratory data estimated the number of BC women (assigned female at birth or unknown sex) aged 13–49 who received routine prenatal serological screening (HIV, hepatitis B, syphilis and rubella) from 2008–2019. Anti-HCV tests ordered the same day as routine prenatal screens were considered prenatal anti-HCV tests. Assessment of follow-up was based on HCV RNA and/or genotype testing within one year of new prenatal anti-HCV diagnoses.

**Results:**

In 2019, 55,202 routine prenatal screens were carried out for 50,392 BC women. Prenatal anti-HCV tests increased significantly, from 19.6% (9,704/49,515) in 2008 to 54.6% (27,516/50,392) in 2019 (p<0.001). New prenatal anti-HCV diagnoses (HCV positive diagnoses at first test or seroconversions) declined from 14.3% in 2008 to 10.1% in 2019. The proportion of women with new prenatal anti-HCV diagnoses that were a result of a first HCV test declined from 0.3% (29/9,701) in 2008 to 0.03% (8/27,500) in 2019. For women known to be anti-HCV positive at the time of prenatal screening, the proportion who had a prenatal anti-HCV test increased from 35.6% in 2008 to 50.8% in 2019.

**Conclusion:**

Prenatal anti-HCV testing increased substantially over the study period. However, new HCV diagnoses remained relatively stable, suggesting that a considerable proportion of BC women with low or no risk are being screened as part of prenatal care. The vast majority of women with new HCV diagnoses receive appropriate follow-up HCV RNA and genotype testing, which may indicate interest in HCV treatment. These findings contribute to the discussion around potential for prenatal anti-HCV screening in an effort to eliminate HCV.

## Introduction

Hepatitis C virus (HCV) affects approximately 250,000 people in Canada, among whom 39% are women [1,2]. Because about 75% of acute infections progress to chronic HCV infection and most remain asymptommatic for decades, it is estimated that up to half of Canadians living with HCV are unaware of their infection [1]. Left untreated, chronic HCV infection can lead to cirrhosis, liver cancer, and death [3]. For women of childbearing age, there is the additional concern of HCV in pregnancy and vertical transmission to their babies. Between 2007–2016, HCV infection rates among women in Canada aged 20–39 were over 30% higher than women over age 40 [2], and available models estimate 3,500 HCV-affected women become pregnant each year [4,5]. HCV has been associated with pregnancy complications, including gestational diabetes, intrahepatic cholestasis, preterm labour and delivery, low birth weight, and miscarriage [6]. Vertical HCV transmission affects 4–7% of infants born to pregnant women living with chronic HCV and up to 11% of infants born to those with HIV-HCV coinfection [7]. It is estimated that half of infants born with HCV will become chronically infected–potentially leading to pediatric cirrhosis, liver cancer, and impaired quality of life [8,9].

Fortunately, HCV-related harms can be prevented with highly effective and tolerable direct acting antiviral therapies (DAAs). DAAs achieve cure in >95% of people treated and, as of 2015, are widely available through Canada’s universal healthcare system, signifying an exciting shift away from arduous and less effective interferon-based treatments. The advent of DAAs has inspired the possibility of eliminating the virus nationally and globally by 2030 [1]. According to the World Health Organization, this will require that 90% of people living with HCV are diagnosed [1]. In British Columbia (BC), public health efforts to scale up HCV testing and linkage to DAA treatment providers have generated optimism in meeting the 2030 goal [10]. Yet, as of 2018, an estimated 25% of people living with HCV in BC remain undiagnosed, and 13% who previously tested anti-HCV positive have not received confirmatory testing [10]. Research from the United States (US) suggests that, due to a combination of structural barriers, gendered vulnerabilities, and rising rates of injection drug use, HCV prevalence is increasing among younger women of childbearing age who are less engaged in the healthcare system [11].

Current Canadian guidelines recommend anti-HCV testing based on risk (e.g. past or current injection drug use, incarceration history, unregulated tattoos/piercings, exposure to contaminated blood products, or exposure within HCV endemic countries) for women of childbearing age to identify those who are undiagnosed and to follow-up with confirmatory RNA testing, genotype testing, and linkage to treatment [1]. In line with these guidelines, the Society of Obstetricians and Gynaecologists of Canada also recommends targeted HCV screening for pregnant women with known risk [12]. Clinical experts in hepatology have pointed out limitations of risk-based anti-HCV screening for pregnant women, as many prenatal care providers may be unaware of HCV risk factors or incorrectly perceive patients as low risk, and because patients may be reticent to disclose highly stigmatized risk factors [6,13,14]. Pregnant women who experience higher risk for HCV due to past or current substance use may delay or avoid seeking prenatal care because of stigma within healthcare towards people who use drugs and the possibility of their children being apprehended due to child welfare concerns [15]. Current testing recommendations may also contribute to missed opportunities to re-engage pregnant women who were previously diagnosed HCV antibody and/or RNA positive but did not receive HCV treatment due to past eligibility restrictions, were excluded for other reasons, or opted not to initiate treatment. Indeed, previous studies have suggested that risk-based prenatal anti-HCV screening under-estimates prenatal HCV prevalence and misses opportunities to engage women into HCV care [16]. DAAs are still contraindicated during pregnancy, and there is no effective strategy to prevent vertical transmission [17]. Diagnosis and treatment of HCV among women prior to pregnancy or in pregnancy intervals is essential for engagement into HCV care and to support follow-up HCV testing recommendations for infants [5,18].

Extant literature suggests uptake of prenatal anti-HCV screening and follow-up in Canada is increasing in some jurisdictions. Kuo et al. (2014) demonstrated that in BC, where prenatal anti-HCV screening is risk-based, prenatal anti-HCV testing increased from 17.6% of all prenatally tested women in 2007 to 25.9% in 2011, yielding an average annual prenatal HCV prevalence (antibody and/or RNA positive) of 0.6% [19]. Recently, Biondi et al. (2020) used aggregated public health laboratory data from Ontario to demonstrate that between 2010–2017, risk-based prenatal anti-HCV testing reached just 2.4% of women in the province [20]. Currently, Saskatchewan is the only province in Canada offering universal prenatal anti-HCV screening.

Taken together, this evidence has led to calls for adding anti-HCV tests to universal prenatal serological screens (HIV, hepatitis B (HBV), syphilis, and rubella)–which are provided on an ‘opt-out’ basis for all pregnant women in BC [5]. Arguments for universal prenatal anti-HCV screening are that identifying women living with HCV before or in between pregnancies is critical to reaching national HCV elimination goals and optimizing the health and well-being of parents and their children, with potential to prevent vertical HCV transmission [21–23]. Arguments to continue risk-based prenatal anti-HCV testing are that the additional cost of universal prenatal anti-HCV screening may outweigh the benefit of identifying a small number of anti-HCV positive women, especially as HCV prevalence continues to decline in BC [10,22]. Exploring the utility of prenatal HCV screening for case detection and linkage to care in the era of DAAs is important not only because HCV elimination is within BC’s reach, but also because it may inform women and children’s health services and programming.

Using BC population-level laboratory data from 2008–2019, the current study describes uptake of prenatal anti-HCV screening and follow-up testing in BC. Our aims were to 1) calculate the frequency of anti-HCV testing among all women in BC; 2) determine the frequency of prenatal anti-HCV testing; 3) calculate the frequency of new HCV diagnoses and HCV seroconversions following prenatal anti-HCV tests, and; 4) to describe frequency of follow-up testing (HCV RNA and/or genotype testing) among women newly diagnosed with HCV–all women in BC and for those who had a prenatal anti-HCV screen.

## Methods

BCCDC Public Health Laboratory (BCCDC PHL) performs all rubella and syphilis screening, approximately 95% of all HIV screening and all confirmatory HIV testing, and 95% of all HCV antibody and confirmatory RNA testing for the province. In addition, two-thirds of HBV surface antigen screening (HBsAg), and all confirmatory HBV testing is carried out at BCCDC PHL. This provides an accurate testing denominator for the population and confidence in the completeness of testing data to determine new diagnoses and prevalent cases.

Prenatal screens in BC are offered at the first prenatal visit, which could be at any time during pregnancy or at delivery. Tests submitted as part of prenatal screening for HIV, syphilis, rubella and HBsAg are flagged in the BCCDC PHL information system as a prenatal package. Eligible BCCDC PHL test records for this study were people assigned female at birth or with unknown sex, who were aged 13–49 years old at the time of testing, and who received routine prenatal screening in BC between January 1, 2008, and December 31, 2019. In this study, we refer to ‘women’ as being people that were assigned as being female sex at birth; although ‘woman’ also implies gender identity, this was not measured in this study.

Prenatal anti-HCV screens were defined as anti-HCV tests ordered on the same day as prenatal screens. HCV cases were defined via detection of antibodies to HCV (anti-HCV) by a third-generation enzyme immunoassay ADVIA Centaur® HCV (Siemens, Canada). If the primary screen was positive, the specimen was retested by a different manufacturer’s third-generation enzyme immunoassay (Architect anti-HCV, Abbott, Canada). Only specimens positive by both manufacturers’ tests were considered anti-HCV positive. If a female patient record was HCV RNA positive, we assumed that they had a previous anti-HCV test. Prenatal HCV prevalence was defined as the yearly sum of all previously known and new diagnoses among prenatally screened women over the twelve-year study period. Women with repeated anti-HCV tests were those who had more than one anti-HCV test. Previously known HCV diagnosis was defined as women whose anti-HCV positive status was known prior to their prenatal screen; these women were further grouped into those given a routine prenatal screen only and those given a routine prenatal screen plus a prenatal (repeated, and therefore unnecessary) anti-HCV test. We assessed frequency of repeated prenatal HCV testing among women who had prenatal screens by counting the yearly number of women who were previously known to be HCV positive prior to their prenatal screen and received routine prenatal screen only (no prenatal HCV test), and the number of women who were known to be HCV positive prior to their prenatal screen (based on their testing records) and received a prenatal screen plus a prenatal anti-HCV test (repeated unnecessary HCV test), and dividing each count by the annual number of women who were anti-HCV positive at time of prenatal screen.

Women with new HCV diagnoses were stratified into those that were an anti-HCV positive at first test and seroconversions. Women who were anti-HCV positive at first test were those with no record of a prior anti-HCV test and who tested positive at their first anti-HCV test. Women who seroconverted to anti-HCV positive were those who had a prior negative anti-HCV test and who tested positive at a subsequent anti-HCV test. Annual HCV seroconversion rates were calculated by dividing the number of women who had seroconverted by the number of women who had previously tested negative. Annual rates of first time reactive HCV diagnoses were calculated by dividing the number of new diagnoses at prenatal screening in a particular year by the total number of individual women prenatally screened for HCV that year. Comparisons were made across age groups and years. We evaluated follow-up HCV testing among women newly diagnosed with a prenatal HCV screen and among all other women aged 13–49 who were newly diagnosed in BC by creating a testing ‘flag’ for anti-HCV, RNA PCR, and genotype tests that occurred within one year after a new positive anti-HCV diagnosis.

Prenatal test data were ordered by either date of collection or receipt at BCCDC PHL and by patient-year, then deterministically linked to HCV test data by a combination of name, public health number, and date of birth. A unique study identification number was assigned to each person. Test uptake was the percentage of women tested each year evaluated by the year of specimen collection; thus, prenatal and anti-HCV tests were counted once for each woman in any given year. If a woman had multiple testing episodes within a calendar year, the most recent specimen results were used. Test data were therefore grouped by patient and year, though women were counted only once per year, they could appear more than once in the study period if they had multiple prenatal screens in different years. Similarly, maximum age and reactive status were counted once for each woman in a given year. Each woman was therefore unique in a given calendar year, but individual women could repeat across years. Analysis for this paper was carried out using SAS software 9.4©.

### Ethics statement

The *BC Public Health Act* provides legal authority to the BCCDC PHL to steward and link laboratory and surveillance data for communicable disease surveillance and a mandate to conduct public health evaluations. As this analysis was performed as BC pubic health surveillance, ethics approval was not required. BCCDC PHL surveillance data are de-identified and analyzed anonymously; thus, informed consent was not sought.

## Results

Table 1 displays data for all women in BC aged 13–49 who had anti-HCV testing, the number who had more than one anti-HCV test (repeated tests), new diagnoses (positive anti-HCV diagnoses at first test, and seroconversions), and follow-up testing from 2008–2019. The annual number of women who had an anti-HCV test increased by 123%, from 46,877 in 2008 to 104,897 in 2019; and the number of women who had more than one prior (repeated) anti-HCV test increased by 200%, from 20,108 in 2008 to 60,222 in 2019. The proportion of women with new anti-HCV diagnoses declined from 1.3% (616/46,877) in 2008 to 0.3% (367/104,897) in 2019. Among newly diagnosed women, the proportion who tested positive at their first anti-HCV test declined from 64% (394/616) to 48.5% (178/367) over the study period. Likewise, the proportion of women who had an HCV seroconversion among those with repeated anti-HCV tests declined from 1.1% (189/20,108) in 2008 to 0.3% (189/60,222) in 2019, representing a 77% relative reduction in seroconversions even with an increase in repeated testing of three times between 2008 and 2019. In 2008, 89.8% (553/616) of women newly diagnosed anti-HCV positive were followed up with HCV RNA or genotype testing, which was 83.6% (363/434) in 2018. The proportion was lower for 2019 (75.7% (278/367)), likely due to truncation bias as follow-up time was shorter to assess RNA and genotype testing after antibody testing.

**Table 1:** Anti-HCV testing, new diagnoses, and follow-up testing (HCV RNA and/or genotype) among women aged 13–49 in BC, 2008–2019.

Year	Unique women tested for anti-HCV*	Women with repeated anti-HCV tests	New HCV diagnoses** n (%)	Anti-HCV positive diagnoses at first test*** n (%)	HCV seroconversion^ n (%)	Follow-up HCV RNA and genotype testing*** n (%)
2008	46,877	20,108	616 (1.3)	394 (64.0)	222 (1.1)	553 (89.8)
2009	49,191	21,655	552 (1.1)	351 (63.6)	201 (0.9)	497 (90.0)
2010	50,797	23,355	450 (0.9)	293 (65.1)	157 (0.67)	400 (88.9)
2011	53,068	25,289	397 (0.7)	269 (67.8)	128 (0.5)	353 (88.9)
2012	59,948	28,844	415 (0.7)	257 (62.0)	158 (0.5)	385 (92.8)
2013	69,281	33,697	433 (0.6)	249 (57.5)	184 (0.5)	385 (88.9)
2014	74,329	37,609	422 (0.6)	229 (54.3)	193 (0.5)	374 (88.6)
2015	82,965	43,091	500 (0.6)	238 (47.6)	262 (0.6)	445 (89.0)
2016	89,386	48,055	486 (0.5)	249 (51.2)	237 (0.5)	425 (87.4)
2017	93,283	51,588	491 (0.5)	238 (48.5)	253 (0.5)	416 (84.7)
2018	98,140	55,427	434 (0.4)	225 (51.8)	209 (0.4)	363 (83.6)
2019	104,897	60,222	367 (0.3)	178 (48.5)	189 (0.3)	278 (75.7)

*Women were counted the earliest year they were tested.

**Denominator is number of unique women who tested for anti-HCV that year.

***Denominator is number of women with new HCV diagnoses (i.e. no record of a prior anti-HCV test or prior negative anti-HCV test).

^Denominator is number of women with prior negative anti-HCV tests.

**Fig 1:**
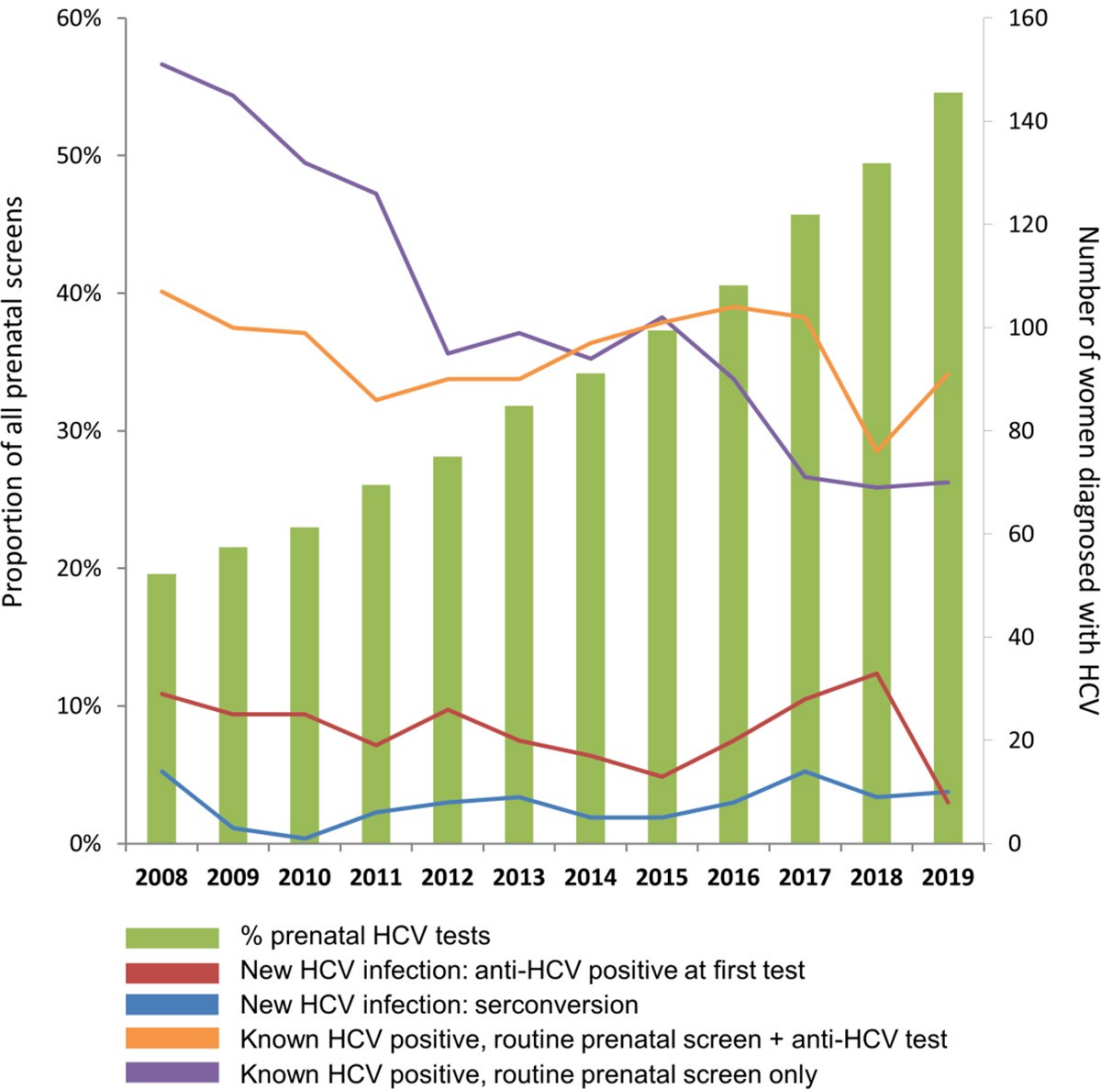
Proportion of all prenatal screens that included an anti-HCV test and number of women diagnosed anti-HCV positive as a result of a prenatal HCV screen, including new diagnoses (positive at first test and seroconversion) and previously known diagnoses (repeat testers) in BC, 2008–2019.

Table 2 presents the annual frequency of prenatal anti-HCV testing among BC women aged 13–49 who had a routine prenatal screen from 2008 to 2019, including prenatal HCV prevalence and the proportional contribution of previously known and new HCV diagnoses. Trends are graphically represented in Fig 1. The number of unique women who had routine prenatal screening in BC was relatively constant over the study period, with about 50,000 screened each year, exceeding the annual number of deliveries in the province reported by Perinatal Services BC [24]. The mean age of women prenatally screened increased from 29.6 years in 2008 to 31.5 years in 2019. The proportion of routine prenatal screens that included a prenatal anti-HCV test increased significantly over the study period, from 19.6% (9,704/49,515) in 2008 to 54.6% (27,516/50,392) in 2019 (p<0.001) (Fig 1 green bars). Additionally, the number of women who had repeated anti-HCV tests increased by 329%, from 3,628 in 2008 to 15,555 in 2019. HCV prevalence among women who had routine prenatal screening declined from 0.61% (301/49,512) in 2008 to 0.36% (179/50,392) in 2019, representing a 41% change.

**Table 2:** Prenatal screening, prenatal anti-HCV testing, new diagnoses (positive at first test or seroconversions), and follow-up testing (RNA and/or genotype) among women aged 13–49 in BC, 2008–2019.

	Unique women receiving routine prenatal screens in each year N	Mean Age (sd)†	Routine prenatal screens + anti-HCV tests n (%)*	Women with repeated anti-HCV tests	Prenatal HCV prevalence ** n (%)	Known HCV positives, routine prenatal screen only*** n (%)	Known HCV positives, routine prenatal screen + anti-HCV test*** n (%)	New HCV diagnoses*** n (%)	Anti-HCV positive diagnoses at first test ** n (%)	HCV seroconversions^ n (%)	Follow-up HCV RNA and genotype testing^^ n (%)
2008	49,515	29.6 (5.84)	9,704 (19.6)	3,628	301 (0.61)	151 (50.2)	107 (35.6)	43 (14.3)	29 (0.3)	14 (0.39)	35 (81.4)
2009	48,715	29.8 (5.77)	10,488 (21.5)	4,067	273 (0.56)	145 (53.1)	100 (36.6)	28 (10.3)	25 (0.24)	3 (0.07)	22 (78.6)
2010	48,178	29.9 (5.72)	11,064 (23.0)	4,516	257 (0.53)	132 (51.4)	99 (38.5)	26 (10.1)	25 (0.22)	1 (0.02)	24 (92.3)
2011	48,873	30.1 (5.68)	12,736 (26.0)	5,437	236 (0.48)	126 (53.4)	85 (36.3)	25 (10.6)	19 (0.15)	6 (0.11)	23 (92)
2012	49,221	30.3 (5.6)	13,843 (28.1)	6,019	219 (0.44)	95 (43.4)	90 (41.1)	34 (15.5)	26 (0.19)	8 (0.13)	33 (97.1)
2013	48,873	30.4 (5.52)	15,544 (31.8)	7,074	218 (0.45)	99 (45.4)	90 (41.3)	29 (13.3)	20 (0.13)	9 (0.13)	27 (93.1)
2014	49,770	30.6 (5.45)	17,011 (34.2)	8,148	213 (0.43)	9 (44.1)	97 (45.5)	22 (10.3)	17 (0.10)	5 (0.06)	20 (90.9)
2015	50,145	30.8 (5.42)	18,712 (37.3)	9,190	221 (0.44)	102 (46.2)	101 (45.7)	18 (8.1)	13 (0.07)	5 (0.05)	18 (100)
2016	50,783	30.9 (5.32)	20,609 (40.6)	10,809	222 (0.44)	90 (40.5)	104 (46.9)	28 (12.6)	20 (0.10)	8 (0.07)	26 (92.9)
2017	50,815	31.1 (5.31)	23,232 (45.7)	12,481	215 (0.42)	71 (33.0)	102 (47.4)	42 (19.5)	28 (0.12)	14 (0.11)	40 (95.2)
2018	50,418	31.3 (5.3)	24,923 (49.4)	13,842	187 (0.37)	69 (36.9)	76 (40.6)	42 (22.5)	33 (0.13)	9 (0.07)	39 (92.9)
2019	50,392	31.5 (5.24)	27,516 (54.6)	15,555	179 (0.36)	70 (39.1)	91 (50.8)	18 (10.1)	8 (0.03)	10 (0.06)	16 (88.9)

†sd: standard deviation.

*Denominator is number of unique women receiving routine prenatal screens.

**Denominator is number of women receiving routine prenatal screen plus anti-HCV prenatal test.

***Denominator is number of women anti-HCV positive at time of prenatal screen.

^Denominator is number of women with prior negative anti-HCV tests.

^^Denominator is number of women with new HCV diagnoses.

For women who were already known to be anti-HCV positive at the time of their routine prenatal screen, the proportion who received a routine prenatal screen declined from 50.2% (151/301) in 2008 to 39.1% (70/179) in 2019 (Fig 1, lilac line), while the proportion who had a routine prenatal screen plus a prenatal anti-HCV test increased from 35.6% in 2008 to 50.8% in 2019 (Fig 1, orange line).

The median annual number of women who had a new HCV diagnosis at the time of their prenatal anti-HCV screen over the study period was 28 (range: 18–43), with the lowest number reported in 2019 (n = 18). The proportion who had an anti-HCV positive diagnosis at first test as a result of a prenatal anti-HCV test declined from 0.3% (29/9,701) in 2008 to 0.03% (8/27,500) in 2019 (Fig 1, red line). As the number of prenatally screened women who had repeated anti-HCV tests increased, the rate of anti-HCV seroconversion in this group declined from 0.39% (14/3,628) in 2008 to 0.06% (10/15,555) in 2019 (Fig 1, blue line). Compared to other women in the province, higher proportions of women who were newly diagnosed anti-HCV positive after a prenatal test received follow-up RNA and/or genotype HCV testing within one year of their diagnosis.

## Discussion

Using population surveillance and laboratory data, this study described uptake of prenatal HCV screening, subsequent HCV case detection, and follow-up HCV RNA/genotype testing among women in BC from 2008–2019. Current HCV testing and treatment guidelines in Canada recommend that clinicians assess patient’s exposure risk prior to testing for HCV [12,25]; yet, we observed a steep rise in the proportion of women who received a prenatal anti-HCV test along with routine prenatal serology, reaching 54.6% in 2019. Women whose anti-HCV diagnosis was known before their prenatal HCV screen comprised the majority of prevalent prenatal HCV cases, and yet, each year, a median of 28 women were newly diagnosed anti-HCV positive as a result of prenatal HCV testing. We observed an increase in unnecessary prenatal anti-HCV testing among women previously diagnosed anti-HCV positive, accounting for over half of those who were anti-HCV positive at the time of their prenatal screen. Follow-up HCV RNA and genotype testing were consistently high among newly diagnosed women after prenatal anti-HCV testing but lower among all other women newly diagnosed not through prenatal screening in the province, suggesting that some may require additional support to engage in HCV treatment.

While the number of women who received routine prenatal screening in BC was consistent over the 12 year study period, the significant increasing trend of prenatal anti-HCV testing may indicate that more are being assessed and identified as being at-risk. The increase may also indicate that the widely publicized roll-out of DAAs within Canada’s publically funded health insurance system in 2015 resulted in increased awareness of HCV and subsequent anti-HCV screening. In addition, we observed that as more women in BC had prenatal anti-HCV testing, anti-HCV prevalence in this group declined overall (from 0.60% in 2008 to 0.34% in 2019), suggesting that more lower or no-risk women are being screened. Other settings with risk-based prenatal screening have reported markedly higher prenatal anti-HCV prevalence, lending greater weight to calls for universal prenatal anti-HCV testing. For example, in the US, using a nationally representative sample, Schille et al. (2018) reported that risk-based prenatal anti-HCV testing reached 13.4% of pregnant women who had live births in 2015, yielding 3.6% prenatal HCV prevalence [26]. These results and others informed support for universal prenatal anti-HCV screening by the American Association for the Study of Liver Diseases and Infectious Diseases Society of America [27]. This was followed by the Center for Disease Control’s recent recommendation (March 2020) to screen all pregnant women during each pregnancy in settings where prevalence of chronic HCV infection is >0.1% [28]. Important epidemiological and jurisdictional differences may explain higher HCV prevalence among prenatal women in the US. HCV testing in BC is estimated to be 3.5-fold higher than in the US, and our finding related to the declining number of positive tests among prenatally screened women in BC suggests that more who are at low-risk are being tested and/or that fewer women who are pregnant or planning pregnancy are at risk of HCV infection. Moreover, several population-based studies in the US have indicated that rising maternal and pediatric HCV prevalence is likely related to concomitant increasing opioid use among women of childbearing age [6,29–31]. Future exploration into differences between women in BC who had a prenatal anti-HCV test vs. those who did not will help better understand the testing trends we have found in this study, especially to evaluate the utility of risk-based testing guidelines when it appears that more healthcare providers are opting to test women with lower risk.

Among prenatally screened women who were anti-HCV positive, the majority had a previous positive anti-HCV test on record. Repeated anti-HCV testing with a prenatal HCV screen was carried out for 43% of the women who had been previously diagnosed anti-HCV positive, suggesting that many care providers–and perhaps the women themselves–were unaware of their anti-HCV positive status before to their prenatal screen. Alternatively, this may signal that some care providers are unaware that anti-HCV positive status does not change, and that repeated anti-HCV testing after a positive result is unnecessary. Repeated anti-HCV testing in this study is higher than what was reported in BC from 2007–2011 by Kuo et al. (2014), where 38.5% of women with known prior HCV infection were again tested with an anti-HCV prenatal screen [19]. These findings could indicate the efficacy of prenatal anti-HCV screening in identifying new HCV diagnoses among women with established risk factors who may have been missed by other targeted testing strategies. Nevertheless, Canadian and BC testing guidelines are clear that because an anti-HCV positive status does not change, subsequent anti-HCV testing is unnecessary and burdensome for both patients and the healthcare system; instead, healthcare providers should follow-up with confirmatory RNA and genotype testing, and treatment initiation when indicated [3]. Repeat testing in our study was similar to findings from Biondi et al. (2020), where 42% of prenatal anti-HCV positive tests in Saskatchewan were subsequently repeated [20]. Conversely, we found that the number of women living with HCV who were not given a repeat anti-HCV test with their prenatal screen declined from 2008–2016, and levelled off from 2017–2019. This trend may signal that in the DAA treatment era, more prenatal care providers are becoming familiar with HCV infection and testing guidelines, including following up with confirmatory RNA and genotype testing. Using tools to prevent unnecessary repeated testing, such as electronic health records, are essential to reduce both over testing women and also costs to the healthcare system. BCCDC PHL recently implemented a new HCV testing algorithm that reflexively tests HCV RNA for all anti-HCV positive test results, which will presumably reduce unnecessary repeat anti-HCV testing among people who have previously tested anti-HCV positive. Future work will assess the HCV care cascade for women who are prenatally diagnosed anti-HCV positive, including treatment uptake in between pregnancies, vertical transmission, and pediatric HCV follow-up care.

This study demonstrated that from 2008–2019, 9.8% (263/2,676) of prevalent prenatal HCV diagnoses were among women whose prenatal anti-HCV screen was their first HCV test and 3.44% (92/2,676) were among women who had seroconverted to anti-HCV positive. We were not able to confirm pregnancy in this study; yet, HCV diagnoses subsequent to prenatal HCV testing may signal missed chances to screen before pregnancy and provide holistic obstetric care for parents and their infants [5]. These HCV diagnoses may have also been among women presenting late for prenatal care, or among women who received no prenatal care. Secondary benefits of diagnosing HCV in pregnancy may include identifying and supporting parents living with HCV and experiencing substance use and/or other social or structural barriers to healthcare [21,23]. In a cohort study of >800 pregnant women accessing an obstetric clinic specializing in substance use treatment in Boston, US, gaps in the HCV care cascade were highlighted at the RNA and genotype testing and treatment initiation stages [32]. The study authors concluded that while HCV testing and treatment uptake improved with the introduction of DAA therapies, wraparound health and social services were required to support more pregnant women living with HCV infection and their infants to engage further with the HCV care cascade. In the present study, we found that the vast majority of women in BC who were newly diagnosed HCV positive after a prenatal HCV screen received follow-up RNA and/or genotype testing–suggesting that they are being closely monitored and linked to HCV care in the province. In comparison, there was a steady increase in the proportion of all other newly diagnosed women of reproductive age in BC who were lost to follow-up–reaching 24.3% in 2019 from 10.2% in 2008. Outside of pregnancy, HCV infection is a significant concern to women’s health. As BC works to reach the 2030 HCV elimination goals, considering women of childbearing age as a key population will require tailored policy and programming that supports to access HCV testing and to initiate treatment. Further work is needed to better understand the long-term health, social, and economic benefits of risk-based vs. universal prenatal screening and to assess the HCV care cascade for mothers and their infants in BC.

### Limitations

Using BCCDC PHL data, we were unable to confirm pregnancy among prenatally screened women, and therefore cannot comment on risk for vertical transmission or other outcomes related to HCV in pregnancy. In addition, there may be some misclassification bias in our study due to data entry errors, specifically date of birth and sex assigned at birth. BCCDC PHL data does not contain information about gender identity, and therefore we cannot comment on prenatal anti-HCV screening uptake among people who were classified as being female sex assigned at birth, but do not identify as women. We recognize that transgender men and other gender-diverse people may experience unique barriers to prenatal anti-HCV screening and linkage to HCV care. Future work should focus on the specific HCV and prenatal care needs of this key population. We extracted the data in April 2020 and defined follow-up HCV RNA and genotype testing within one year of a positive anti-HCV test result. Thus HCV RNA and genotype follow-up testing for 2019 may be affected by truncation bias. Finally, BCCDC PHL is missing about 5% of HCV and HIV tests, and about 30% of HBV tests in the province.

### Conclusion

We have demonstrated that prenatal anti-HCV testing identifies new cases of HCV infection among women of childbearing age in BC and provides opportunities to follow-up with confirmatory testing and linkage to care. It follows that identifying women living with HCV within prenatal care settings may support HCV treatment between pregnancies, as well as sustained infant testing and care. Moreover, as the first step in the HCV care cascade, prenatal anti-HCV testing offers chances to engage families in health promotion and harm reduction programming as well as other community-based resources. BC’s prenatal HCV prevalence continues to decline; however, as we have demonstrated, testing trends continue to rise. Further discussions about the additional utility of universal prenatal HCV screening for women’s health overall and women experiencing heightened risk for infection or transmission are warranted.
